# Evaluation of ^11^C-Acetate and ^18^ F-FDG PET/CT in mouse multidrug resistance gene-2 deficient mouse model of hepatocellular carcinoma

**DOI:** 10.1186/s12880-015-0058-z

**Published:** 2015-05-16

**Authors:** Paul R. Territo, Mary Maluccio, Amanda A. Riley, Brian P. McCarthy, James Fletcher, Mark Tann, Romil Saxena, Nicholas J. Skill

**Affiliations:** 1 Department of Surgery, Indiana University School of Medicine, C519 Walthur Cancer Research Building (R3), 980 W Walnut Street, Indianapolis, IN 46077 USA; 2 Department of Surgery, Radiology and Imaging Sciences, Indianapolis, IN 46202 USA

## Abstract

**Background:**

Hepatocellular carcinoma (HCC) remains a global health problem with unique diagnostic and therapeutic challenges, including difficulties in identifying the highest risk patients. Previous work from our lab has established the murine multidrug resistance-2 mouse (MDR2) model of HCC as a reasonable preclinical model that parallels the changes seen in human inflammatory associated HCC. The purpose of this study is to evaluate modalities of PET/CT in MDR2^−/−^ mice in order to facilitate therapeutic translational studies from bench to bedside.

**Methods:**

^18^F-FDG and ^11^C-acetate PET/CT was performed on 12 m MDR2^−/−^ mice (n = 3/tracer) with HCC and 12 m MDR2^−/+^ control mice (n = 3/tracer) without HCC. To compare PET/CT to biological markers of HCC and cellular function, serum alpha-fetoprotein (AFP), lysophosphatidic acid (LPA), cAMP and hepatic tumor necrosis factor α (TNFα) were quantified in 3-12 m MDR2^−/−^ (n = 10) mice using commercially available ELISA analysis. To translate results in mice to patients ^11^C-acetate PET/CT was also performed in 8 patents suspected of HCC recurrence following treatment and currently on the liver transplant wait list.

**Results:**

Hepatic^18^F-FDG metabolism was not significantly increased in MDR2^−/−^ mice. In contrast, hepatic ^11^C-acetate metabolism was significantly elevated in MDR2^−/−^ mice when compared to MDR2^−/+^ controls. Serum AFP and LPA levels increased in MDR2^−/−^ mice contemporaneous with the emergence of HCC. This was accompanied by a significant decrease in serum cAMP levels and an increase in hepatic TNFα. In patients suspected of HCC recurrence there were 5 true positives, 2 true negatives and 1 suspected false ^11^C-acetate negative.

**Conclusions:**

Hepatic ^11^C-acetate PET/CT tracks well with HCC in MDR2^−/−^ mice and patients with underlying liver disease. Consequently ^11^C-acetate PET/CT is well suited to study 1) HCC emergence/progression in patients and 2) reduce animal numbers required to study new chemotherapeutics in murine models of HCC.

## Background

Hepatocellular carcinoma (HCC) is a primary cancer of the liver that most often develops in identifiable patients with underlying liver disease, like hepatitis. HCC is the fourth most common cancer in the world with age-normalized incidence rates of 2.1 per 100,000 in North America [1, 2]. The risk of HCC is thought to be associated with inflammatory changes within the liver microenvironment which extends over a protracted period of time, with cirrhosis and chronic hepatitis (B and C) accounting for approximately 50 % of all HCC [3]. At our institution, we have a patient population with increased risk that present for treatment before emergence of HCC. This puts our institution in a unique position to evaluate biomarkers of cancer risk along with novel diagnostic tests that would improve stratification in patients with the highest likelihood of cancer, thus resulting in improved diagnostic and treatment paradigms to be applied. Our ability to impact patient outcomes in HCC rests in three key areas: 1) biomarkers of cancer risk, 2) improvements in diagnostic imaging, and 3) improved therapeutic options. This manuscript focuses on the first two issues and investigates the use of lysophosphatidic acid (LPA) profile analysis and PET/CT imaging in the multiple drug resistance-2 (MDR-2) knockout mouse model of HCC.

MDR2 is a membrane-associated protein linked to lipid transportation and is increased in HCC cell lines and tumors [4, 5]. Previous studies have demonstrated *in vitro* MDR2 expression is increased in response to chemotherapeutic agents and that HCC develops spontaneously in MDR2^−/−^ mice [6–11]. More recently we have shown that HCC tumor burden can be reduced by administration of the commercially available LPA biosynthesis/signaling inhibitor BrP-LPA [6] and confirmed the use of MDR2^−/−^ mice as a model for clinical pathologies [12–16]. This current study builds on our use of macroscopic measurements of HCC as indicators of disease progression, and evaluates non-invasive markers and imaging in MDR2^−/−^ mice in order to validate tools pursuant to accurately measuring response to chemotherapeutic agents in future studies.

Currently, the first line therapy for patients not eligible for resection or liver transplantation is sorafenib chemotherapy [17]. However, sorafenib is associated with a meager improvement in overall survival compared to supportive care alone. Recent reports have shown resistance to sorafenib in liver cells linked to phenotypic changes consistent with advanced invasion [18], and that sorafenib response is related to deregulation of mitochondria fusion-related protein optic atrophy 1 (OPA1) and reduction in Phosphatase and tensin homolog (PTEN) expression [19, 20]. The documented stable disease rate associated with sorafenib suggests that it would perhaps be more appropriate as a chemoprevention agent rather than treating established disease. To facilitate these studies an accurate suite of modalities to evaluate pre-clinical therapeutic response *in vivo* is required, including methods to evaluate tumor burden and response to treatment. Accordingly, to better understand the MDR2^−/−^ model of HCC, MDR2^−/−^ mice underwent testing for: 1) HCC biomarker serum alpha fetoprotein (AFP) ELISA; 2) oxidative metabolism by ^11^C-acetate PET/CT; 3) glycolytic metabolism by ^18^ F-FDG PET/CT; 4) lipid metabolism by lysophosphatidic acid variant profile tandem mass spectroscopy; 5) cellular signaling by circulating cAMP ELISA and 6) inflammatory cytokine modulation by hepatic TNFα ELISA. We found that all modalities, except glycolytic metabolism via ^18^ F-FDG PET/CT, differentiated mice with HCC from those without, thus demonstrating potential modalities to monitor HCC development and treatment in MDR2^−/−^ mice. In addition, to confirm the relevance of ^11^C-acetate PET/CT for HCC in mice ^11^C-acetate PET/CT was performed in 8 patients with suspected recurrent HCC following standard of care therapy. We found that 5 of 8 patients were true positives, 2 of 8 were true negatives, and one false negative patient. This information confirms the potential of ^11^C-acetate PET/CT in murine studies to develop novel and new therapies of treatment.

## Methods

### Animals

#### MDR2^−/−^ Mice

All studies were carried out in accordance with, and approval from, the Institutional Animal Care and Use Committee of Indiana University School of Medicine, the U.S. Department of Agriculture’s Animal Welfare Act (9 CFR Parts 1, 2, and 3) and the Guide for the Care and Use of Laboratory Animals [21]. Multiple drug resistance gene null mice were purchased from Jackson Labs, MA (FVB.129P2-^Abcb4tm1Bor/J^) and breeding colonies were established within the Indiana University Laboratory Animal Research Centre. MDR2^−/−^ mice originating from the Friend virus B-type/N (FVB) background have been found to develop HCC more efficiently then mice from C57BL6 background [22]. Mice were maintained on Teklad Lab Animal Diet TD 2014 (Harlan Laboratories USA) with *ad libitum* access to tap water, and a 12:12 (light:dark) hour photoperiod at 22-24 °C. MDR2^−/−^ mice lack the ability to secrete phospholipids into the bile from the liver and they develop degenerative liver disease, fibrosis and portal inflammation. At an early age MDR2^−/−^ mice are used as a model of primary sclerosing cholangitis (PSC) and that treatment with β-blockers reduce fibrosis [23]. PSC is an underlying etiology of HCC in patients. HCC develops in MDR2^−/−^ at approximately 1 year. To date, the precise mechanism connecting MDR2 to fibrosis is not yet known; however, recent studies suggest that NADPH oxidases NOX4 may play a role [24]. Mice were distributed in to three groups for 1) analysis of serum and tissue markers of HCC (n = 10), 2) for ^11^C-acetate PET/CT imaging (n = 3) and 3) for 18 F-FDG PET/CT (n = 3).

Expression of the mutant MDR2 gene was confirmed by PCR of tail DNA samples. Mutant and wild-type primers are described by the Jackson Labs (CGG CGA GGA TCT CGT CGT GAC CCA and GCG ATA CCG TAA AGC ACG AGG AAG and GCT GAG ATG GAT CTT GAG and GTC GAG TAG CCA GAT GAT GG, respectively). PCR reactions for wild type and mutant MDR2 gene were performed separately, but with identical amplification conditions (i.e. Melting 94 °C 30 sec, appealing 59 °C 1 min, elongation 68 °C 1 min × 25). Products were separated on 1 % agarose TBE ethidium bromide gels and visualized under UV illumination. FVB wild type mice were also purchased form Jackson labs (FVB/NJ) and were used as controls.

#### Analytical procedures

##### Alpha Feto Protein (AFP)

Serum AFP levels were quantified in 3-18 M MDR2^−/−^ and MDR2^−/+^ mice using a commercial ELISA (USCN Life Science Inc. Wuhan) as per manufactures instructions.

##### Cyclic Adenosine Mono Phosphate (cAMP)

Serum from 3 to 18 M MDR2^−/−^ and MDR2^−/+^ mice were quantified for cAMP using a commercially available ELISA kit (Invitrogen, CA) as per manufacturer’s instructions.

##### Tumor Necrosis Factor Alpha (TNFα)

5 % (w/v) liver homogenates from 3 to 18 M MDR2^−/−^ and MDR2^−/+^ mice were quantified for TNFα using a commercial murine TNFα ELISA (Invitrogen, CA) as per manufacturer’s instructions.

##### LPA MS/MS analysis

Was performed by Covance Laboratories, IN. under a fee for service contract. MS analyses were performed using a API-4000 (Applied Biosystems, Forster City, CA). Typical operating parameters were as follows: collision gas (CAD) 8 units, curtain gas (CUR) 10 psi, ion source gas 1 (GS1) 15 psi, ion source gas 2 (GS2) 35 psi, electrospray voltage 5000 V with positive ion MRM mode or −4200 V with negative ion MRM mode, and a temperature of heater at 500 °C. Multiple reaction monitoring (MRM) mode was used for measurement of LPAs. Negative and positive monitoring ions have been described previously [25]. Samples (10 μL) were loaded through a LC system (Agilent 1100) with an auto sampler. A TARGA C18 5 μM, 2.1 mm ID × 10 mm TR-0121-C185 (Higgins Analytical, Southborough, MA USA) HPLC column was used for the separation of lysophospholipids. The mobile phase A was MeOH/water/NH4OH (90:10:0.1, v/v/v) and the mobile phase B was 5 mM ammonium acetate in MeOH/water (90:10, v/v). The HPLC separations was 12 min/sample using the following scheme: 1) 100 % A for 3 min with a flow rate at 0.2 mL/min; 2) the mobile phase was changed from 100 % A to 100 % B over 2 min with the flow rate increased from 0.2 to 0.8 mL/min; 3) a constant flow rate of 0.8 mL/min for 5 min; 4) the mobile phase was changed from 100 % B to 100 % A in 1 min with the flow rate decreased from 0.8 to 0.2 mL/min; and 5) constant flow rate of 0.2 mL/min for 1 min.

##### Fibrosis scoring

Hepatic sections (5 μm) were Mason Trichrome stained for collagen and fibrosis scoring was quantified using ImageJ software (http://rsb.info.nih.gov/ij/) as described previously [26].

### *In vivo* imaging

#### PET and CT imaging

For glycolysis imaging, conscious mice were injected with 7.5 ± 0.36 MBq of ^18^ F-FDG via tail vein and returned to an isothermal cage for 30 min to allow for conscious tracer uptake and enzymatic trapping. For oxidative phosphorylation imaging, mice were anesthetized (see below) placed on the scanner bed and injected with 6.6 ± 0.79 MBq of ^11^C-acetate via tail vein once the scanner acquisition sequence had initiated. In all cases, anaesthetic induction was achieved with 3-5 % isoflurane gas (balance medical air), where mice were placed on the scanners imaging bed, and aesthetic plane was maintained with 1-3 % isoflurane gas. Dynamic high resolution ^18^ F-FDG and ^11^C-acetate Positron Emission Tomography [27] images were acquired in list-mode for 30 and 60 min, respectively, using the IndyPETIII small animal PET scanner [27] where animals were maintained isothermal by use of a 750 W convective header (P/N VH2 EH1-0020-01, Vornado Inc, USA). At the end of the PET imaging session, the mouse and carbon fiber bed were transferred to small animal computed tomography (CT) imager (R9, GE Healthcare, Inc USA), where whole body CT images were acquired in two bed positions using a tube voltage, current and shutter speed of 55kVp, 1000 mA and 100 ms, respectively. Post-acquisition, ^18^ F-FDG and ^11^C-acetate images were reconstructed using filtered back projection (FBP) according to published methods [28] with a 60 mm field of view into a single static 3D image volume, and dynamic 4D image series (3D image volumes with time), respectively. In all cases, CT images were reconstructed into a calibrated single 3D image volume using FBP and a 4x4 binning according to manufacturer, yielding a final effective isotropic resolution of 0.160 mm.

#### Image analysis

In all cases, CT images were co-registered to ^18^ F-FDG or ^11^C-acetate PET image volumes using a Analyze 11.0 (AnalyzeDirect) software based on the maximum entropy and mutual information algorithm described previously [29]. Manual whole liver segmentation was performed on registered CT image volumes (Analyze 11.0, AnalyzeDirect). To permit kinetic modelling, left ventricular cavities were also manually segmented for ^11^C-acetate studies to provide an image derived arterial input function. Static ^18^ F-FDG PET images were analysed for percent injected dose per gram (%ID/g) according to published methods [30], while dynamic ^11^C-acetate PET were analysed for liver metabolism by applying a 2 compartment kinetic model described previously [31].

#### Human ^11^C-acetate PET/CT

Was performed in accordance with FDA Physician-Sponsored Expanded Access IND #118204 (James W. Fletcher, M.D., Sponsor). Written informed consent to ^11^C-acetate PET/CT was obtained prior to imaging in accordance with Indiana University IRB protocol #1407718337. Children were not included due to increased protection mandated to children by US department of health and human services (45 CFR 46.405). There is a low propensity of HCC in children and the ^11^C-acetate radiation exposure does not provide a direct benefit to the individual subject. ^11^C-acetate was prepared as described by Mock et al. [32]. The ^11^C-Acetate radiopharmaceutical was administered intravenously at a dose of 20–40 mCi (0.74-1.5 GBq). Inclusion criteria: patient’s with suspected clinical recurrence of HCC after initial radiation or surgical treatment, and who were candidates for surgery or systemic therapy depending on the PET/CT ^11^C-acetate identified location and extent of recurrence (Patients with elevated AFP > 200 or rising AFP but without any measureable disease burden). Patients with a prototype lesion(s) based on CT or MRI imaging that have addition lesions of unclear clinical significance because they are sub-centimeter. Patients who meet transplant eligibility for transplant with a diagnosis of cancer based on AASLD criteria, but in whom our current metastatic evaluation is lacking both sensitivity and specificity. Patients with whom clinical variables are worrisome for a greater disease burden than that suggested by our current imaging standards (CT or MRI) (i.e. acute elevation in AFP, portal vein thrombosis, multifocal lesions, tumor size > 4.5 cm). Patients transplanted with cancer or whom the explant shows a greater disease burden than that noted on pre-transplant imaging putting them at a higher risk for cancer recurrence.

Eight patients received ^11^C-acetate from 9/13/13 through 3/17/2014. Image analysis and interpretation followed the general recommendations of the Society of Nuclear Medicine (SNMMI) and the European Association of Nuclear Medicine (EANM) in their procedure guidelines for PET/CT tumor imaging with other agents. A nuclear medicine physician visually evaluated normal bio-distributions and abnormal accumulations. Tracer accumulation in structures that did not take up the tracer physiologically, or accumulations higher than background activity, was considered to be pathological. Clearly demarcated findings with higher tracer uptake were classified as definitely positive for enhanced radiotracer uptake, and thus indicative of malignancy. SUVmax and SUVpeak were obtained in areas judged to represent abnormal uptake as semi-quantitative measures of tracer bio-distribution. In this study measurement of ^11^C-acetate uptake was limited to the liver. Organ specific ^11^C-acetate uptake in humans has previously been described by Seltzer et al. (2004) [33].

#### Statistical analysis

Biochemical and imaging data are presented as Mean ± 1S.E.M. Determinations of parameter estimates for tracer kinetic models by non-linear regression were performed with custom software developed in IDL 7.1 (Visual Information Solutions). Statistical comparison of cohorts for analytes and metabolic parameters were performed using a 2-tailed unpaired Student’s *t*-test using Excel 12.0 (Microsoft, Inc.). In all cases, statistical significance was taken at the p < 0.05 level. Data correlations were determined using Pearson correlation coefficient (two variables are assumed to have a bivariate normal distribution) analysis and Excel 12.0 (Microsoft, Inc). Correlations amongst ^11^C-acetate uptake, serum LPA/AFP and hepatic TNFα was not undertaken.

## Results

### MDR2^−/−^ Mice

**Fig. 1:**
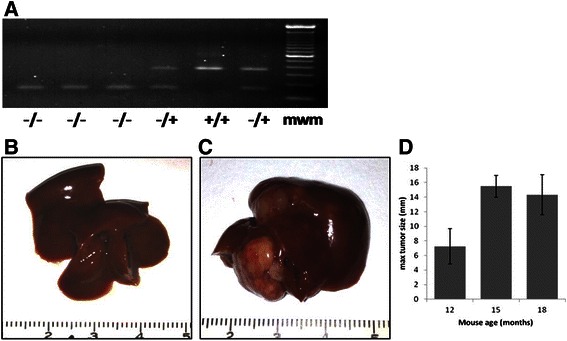
Genotyping of mice and tumor burden in MDR2 mice. **a**) Mouse genotype was confirmed by PCR. 380 bp represented wild type MDR2 gene 180 bp product represents mutant MDR2 gene, mwm equals 100 bp standard. **b**) 12 mo MDR2^−/+^ control mouse liver. **c**) 12 mo MDR2^−/−^ liver and **d**) Tumor burden in MDR2^−/−^ mice 12-18 mo

Mouse genotype was confirmed by RT-PCR (Fig. 1a). There was no difference in body or tissue weights amongst MDR2^−/−^ mice, FVB and MDR2^−/+^ control mice. Hepatic tumors were discovered in 100 % of MDR2^−/−^ (Fig. 1c) mice but were not observed in MDR2^−/+^ (Fig. 1b) mice. Mouse tumor burden increased with age and ranged from 0 mm at 9mo to 7.3 ± 2.4, 15.5 ± 1.5 and 14.33 ± 2.7 mm at 12, 15 and 18mo, respectively (Fig. 1d, Table 1).

**Table 1:** MDR2 mouse characteristics

Mouse strain (n)	Time to HCC development (Months)	Histology	Maximum tumor size (mm)
FVB (10)	N/A	Normal hepatic histology	N/A
MDR2^−/+^ (10)	N/A	Normal hepatic histology	N/A
MDR2^−/−^ (10)	9-12 M	Background liver: Moderate hepatic fibrosis.	12 M – 7 ± 1
		Tumor: Large cells with abundant eosinophilic cytoplasm and prominent central nuclei arranged in a trabecular pattern. Some tumor cells showed evidence of steatosis.	15 M – 15 ± 1
			18 M – 14 ± 2

### Serum AFP

**Fig. 2:**
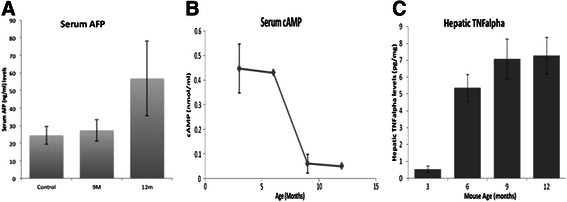
Serum AFP and cAMP and hepatic TNFα are modulated contemporaneously with HCC in MDR2^−/−^ mice. Serum AFP and cAMP and hepatic TNFα were quantified in MDR2^−/−^ mice prior to emergence of HCC at 12 mo. **a**) Serum AFP levels increased in MDR2^−/−^ mice concomitant with the emergence of HCC. **b**) Serum cAMP levels significantly reduced prior to the emergence of HCC. **c**) Hepatic TNFα levels were significantly increased prior to emergence of HCC. D-E) Representative micrographs of MDR2^−/−^ (D) FVB and (E) control mouse livers. Collagen staining (blue) was markedly increased in 12 mo MDR2^−/−^ mice when compared to 12 mo MDR2^−/+^ controls

Serum AFP levels are typically raised in patients with HCC, although the sensitivity and specificity of this biomarker is low [34]. In MDR2^−/+^mice, that don’t develop HCC, serum AFP levels were not significantly increased when compared to FVB controls (Fig. 2a). In contrast, and similarly observed in humans with HCC, serum AFP was significantly (p = 0.048, n = 10) increased in MDR2^−/−^ mice (58 ± 15 ng/ml) when compared to control mice (35 ± 5 ng/ml) (Fig. 2a); however, this increase was not uniform across all mice studied. Serum AFP levels were significantly elevated (40 ± 9 ng/ml) in 63 % of MDR2^−/−^ mice, with little correlation between serum AFP levels and tumor burden (R^2^ = 0.17, n = 10).

### Serum cAMP

Although not measured for clinical diagnosis of HCC, cAMP has previously been linked to HCC [35]. cAMP is a ubiquitous secondary signaling molecule and can be measured using commercially available functional assays. Serum cAMP levels were markedly reduced in MDR2^−/−^ mice as they aged resulting in a significantly reduced circulating level contemporaneous with the detection of HCC. Levels reduced from a high at 3mo of 0.45 ± 0.14 nmol/ml to a low of 0.06 ± 0.03 nmol/ml at 12 m (Fig. 2b).

### Hepatic TNFα

TNFα, an inflammatory cytokine, levels have well correlated with HCC [36]. Murine serum levels of TNFα are typically below detectable levels of commercially available ELISA assays and thus hepatic TNFα levels were quantified directly. Hepatic TNFα levels increased progressively with age in MDR2^−/−^ mice, and increased from low levels of 1 ± 0.2 pg/mg at 3mo to 5.37 ± 0.8, 7.08 ± 1.25 and 7.3 ± 1.1 at 6, 9 and 12mo, respectively (Fig. 2c).

### Fibrosis

There was a markedly increased staining for collagen in livers MDR2^−/−^ mice when compared to MDR2^−/+^ and FVB control mice (Fig. 2D-E). Quantification of staining using ImageJ software showed a 140 % increase over controls.

### HCC histology

The tumors consist of large cells with abundant eosinophilic cytoplasm and prominent central nuclei arranged in a trabecular pattern. Some tumor cells show steatosis. These features are characteristic of hepatocellular differentiation. Distinct from biliary or metastatic tumors, no gland formation is seen.

#### PET/CT Imaging

To non-invasively monitor tumour emergence and growth in MDR2^−/−^, and to advance our knowledge with this modality for future therapeutic work, PET/CT imaging was performed on MDR2^−/−^ and MDR2^−/+^ mice (n = 3).

### ^18^ F-FDG PET/CT

**Fig. 3:**
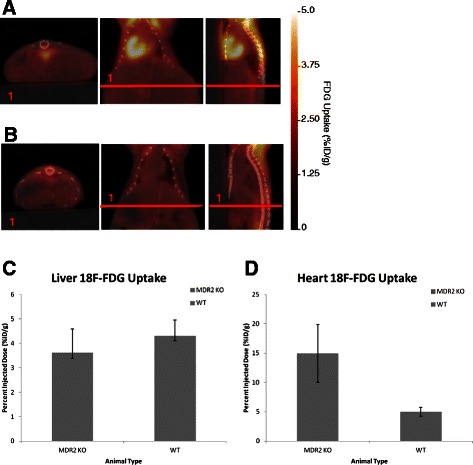
^18^F-FDG PET/CT imaging of MDR2^−/−^, MDR2^−/+^ and FVB wild type mice: PET/CT imaging was performed on 12 mo MDR2^−/−^ and FVB control mice. **a**) Representative ^18^ F-FDG PET/CT parametric (%ID/g) images of MDR2^−/−^ mouse. **b**) Representative ^18^ F-FDG PET/CT parametric (%ID/g) images of FVB control mouse (student’s *t* test p = 0.26, n = 3/group). **c**) Liver ^18^ F-FDG uptake was not significantly greater in MDR2 KO mouse when compared to controls. **d**) Heart ^18^ F-FDG uptake was significantly greater in MDR2 KO mouse when compared to controls (student’s *t* test p = 0.04, n = 3/group)

Clinical monitoring of HCC disease for staging and determination of partial or complete liver transplants have been reported [37–40]. To evaluate the role of glycolytic metabolism in HCC, static ^18^ F-FDG PET/CT scans were conducted on MDR2^−/−^ and control mice. There was no significant difference in ^18^ F-FDG uptake in the livers of MDR2^−/−^ (3.61 ± 0.97 %ID/g) mice when compared to wild type controls (4.3 ± 0.6 %ID/g, p = 0.58, n = 3/group) (Fig. 3a-c). In contrast, cardiac ^18^ F-FDG uptake were significantly higher in MDR2^−/−^ mice when compared to controls (14.9 ± 4.9 vs. 5 ± 0.7, p = 0.05, n = 3/group respectively) (Fig. 3a-b + d). The rationale behind an increase in cardiac ^18^ F-FDG is beyond the scope of this manuscript, but we include the data for dissemination purposes so that others may evaluate the relevance and importance.

### ^11^C-Acetate PET/CT

**Fig. 4:**
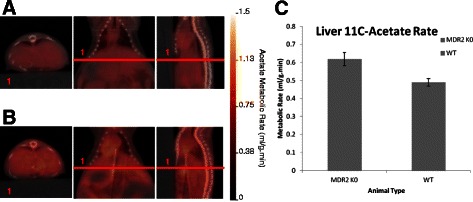
^11^C-acetate PET/CT imaging of MDR2^−/−^, MDR2^−/+^ and FVB wild type mice. ^11^C-acetate PET/CT imaging was performed on 12 mo MDR2^−/−^ and FVB control mice. **a**) Representative ^11^C-acetate PET/CT parametric (ml/g.min) images of MDR2^−/−^ mouse. **b**) Representative ^11^C-acetate PET/CT parametric (ml/g.min) images of FVB control mouse. **c**) Hepatic ^11^C-acetate metabolic rate was significantly greater in MDR2^−/−^ mouse when compared to controls (student’s *t* test p < 0.05, n = 3/group)

Elevated substrate level oxidative phosphorylations, in the absence of glycolytic contributions, have been reported in clinical HCC [37–40]. To monitor substrate level oxidative phosphorylation in HCC, dynamic ^11^C-acetate PET/CT scans were conducted on MDR2^−/−^ and control mice. Hepatic ^11^C-acetate metabolic rates were statistically higher in MDR2^−/−^ (0.62 ± 0.04 ml/g.min) mice when compared to wild-type control mice (0.49 ± 0.02 ml/g.min, p = 0.039, n = 3/group) (see Fig. 4a-c), and no differences were observed between MDR2^−/+^ and FVB control (p > 0.05).

### Serum LPA

**Fig. 5:**
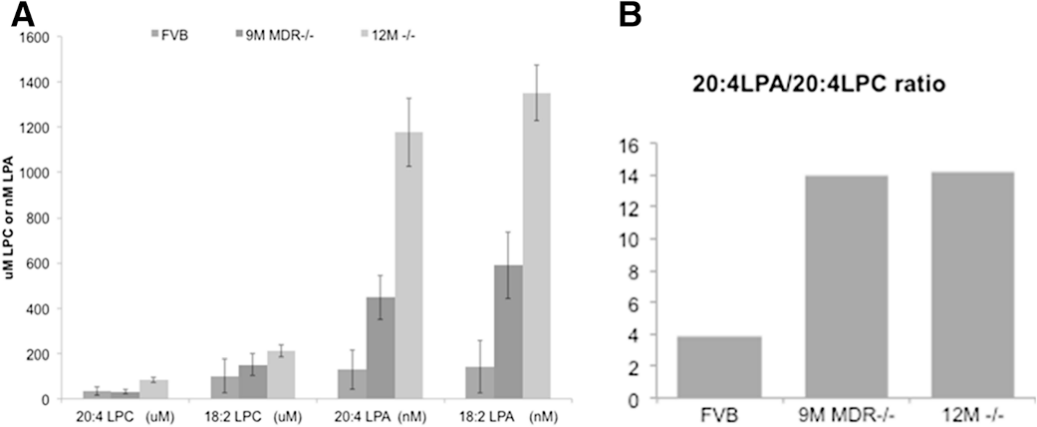
LPA variant profile is modulated prior to HCC emergence in MDR2^−/−^ mice. **a**) Serum levels of LPA and LPC variants were determined by LC/MS. There was a significant increase in 18:2 LPA and 20:4 LPA in MDR2^−/−^ mice prior to the emergence of HCC at 12 mo. LPA variant levels were maximal at 12 mo. **b**) Calculation of the 20:4 LPC/20:4 LPA ratios showed a dramatic change indicative of increased ATX activity

Serum 20:4LPA and 18:2LPA levels where significantly increased in MDR2^−/−^ mice simultaneous with HCC emergence. Levels increased from 129 ± 87nM to 141 ± 116nM in FVB controls to 1177 ± 151 nM and 1331 ± 121nM in 12 m MDR2^−/−^ mice for 20:4LPA and 18:2 LPA, respectively (p < 0.01, n = 10). There was no significant difference in LPA precursor LPC for either variant (p > 0.05, n = 10, Fig. 5a). This increase in LPA in the absence of an increase in precursor LPC demonstrates a definite increase in LPA biosynthesis verified by a marked increase in the LPA to LPC ratio for both 20:4LPA and 18:2LPA (Fig. 5b).

### Human ^11^C-acetate PET/CT

The indication in all except one patient was prior diagnosis of HCC with suspicion for recurrent disease because of rising AFP and indeterminate CT and MRI findings. One patient had rapidly rising AFP but no radiologic abnormality on CT or MRI. Most subjects had had prior treatment with either ^90^Y-radioembolization or external beam therapy. All patients were on waiting list for liver transplant. There were five True positive exam results, two probable True negative exam results and one likely false negative exam result. Following the ^11^C-acetate exam the following actions were initiated: radio embolization with ^90^Y for recurrent disease (n = 1); treatment of recurrent disease with Stereotactic body radiotherapy (n = 1); removal from transplant program list due to unsuspected distant skeletal metastases (n = 1); and continued monitoring (n = 8).

## Discussion

Hepatocellular carcinoma is one of the leading causes of deaths associated with cancer, with greater than 700,000 new cases diagnosed annually, and more than 600,000 deaths worldwide attributed to HCC per annum [41]. Among malignancies, HCC is the fourth most common cancer worldwide [1, 2] with an age-normalized incidence rate of 17, 42, 46, 62, and 371 per 100,000 in the United States, Africa, European Union, South East Asia, and China, respectively [42].

Provided this, there is a distinct need to improve diagnostic and treatment options available for patients with HCC [43]. The potential benefits of ^11^C-acetate PET/CT are numerous. In patients with elevated AFP > 200 or rising AFP but without any measureable disease burden ^11^C-acetate opens up the possibility of detecting occult or subclinical cancer within a high risk diseased liver. In patients with a prototype lesion(s) based on CT or MRI imaging that have addition lesions of unclear clinical significance because they are sub-centimeter ^11^C-acetate imaging opens up the possibility to put sub-centimeter lesions in perspective in patients who otherwise meet imaging criteria for HCC, thereby improving our ability to evaluate overall disease burden. In patients who meet transplant eligibility for transplant with a diagnosis of cancer based on AASLD criteria, but in whom our current metastatic evaluation is lacking both sensitivity and specificity ^11^C-acetate imaging will facilitate improved organ allocation and reduce post-transplant recurrence. In patients in whom clinical variables are worrisome for a greater disease burden than that suggested by our current imaging standards (CT or MRI) (i.e. acute elevation in AFP, portal vein thrombosis, multifocal lesions, tumor size > 4.5 cm) ^11^C-acetate opens up the possibility of improving our understanding of disease burden. In patients transplanted with cancer in whom the explant shows a greater disease burden than that noted on pre-transplant imaging putting them at a higher risk for cancer recurrence ^11^C-acetate imaging may provide some advantage over CT or MRI when used for surveillance in post-transplant patients considered at higher risk for recurrence and in whom clinical trials of novel immunosuppressant protocols post-transplant would become relevant. The MDR2^-/-^ mouse model of HCC provides a murine model whereby new paradigms of HCC diagnosis and targeted therapy can be evaluated [6].

The purpose of this manuscript is to evaluate modalities of PET/CT for monitoring liver disease and HCC in MDR2^−/−^ mice in order to facilitate future studies aimed at exploring experimental treatments. Previous experiments using MDR2^−/−^ mice have relied upon macroscopic measurements of tumor burden [6]; however, using this approach definitive assessments are only feasible at study termination, and therefore do not provide continual monitoring of disease progression and therapeutic response during the emergence and growth of HCC.

In general, monitoring of HCC is challenging, and diagnosis occurs when tissue fibrosis and HCC are advanced. However, HCC emerges in a well-defined cohort of patients with, *inter alia,* Hepatitis C infection (HepC), non-alcoholic steatohepatitis (NASH) or alcoholic liver disease (ALD) cirrhosis, providing an identifiable HCC high-risk group. At Indiana University Hospital patients with HepC, NASH or ALD are generally monitored twice annually for HCC by measurement of AFP, liver biopsy histology, and ultrasonography. Advances in imaging have moved away from the use of ultrasonography and biopsies in favor of PET/CT imaging [37–40, 44]. Moreover, the use of AFP in detection of HCC has proven problematic because of the low sensitivity and selectivity [45]. Given the aforementioned limitations, this manuscript investigates PET/CT analysis of HCC along with serum/tissue biomarkers.

The first objective of this study was to examine current methods of HCC detection, serum AFP and PET/CT, in MDR2^−/−^ mice in order to illustrate compatibility and reproducibility to that seen in patients with HCC. Previous studies have shown that AFP is reduced in response to HCC treatment and that the level of this reduction is predictive of survival [46]. Moreover, PET/CT is used to diagnosis HCC and follow response to treatment [37–40]. To date HCC development has not been demonstrated in MDR2^−/−^ using either AFP or PET/CT.

We found that AFP generally increased contemporaneously with HCC levels in MDR2^−/−^ mice consistent with clinical studies [47], and showed moderate sensitivity and selectivity for HCC. Consequently, serum AFP levels are a reasonable biomarker for HCC in MDR2^−/−^ mice and potentially could be used as a treatment response marker. However, there was a 30 % false negative rate, which argues that additional corroborate evidence would be required. In the clinic, AFP is very helpful for diagnosis and follow-up but negative AFP does not rule out the presence of HCC.

In the current work, ^11^C-acetate PET/CT imaging was shown to be a sensitive marker for monitoring HCC in MDR2^−/−^ mice. This was also seen with patients monitored for HCC reoccurrence [39, 40]. In MDR2^−/−^ mice there was a significant increase in hepatic ^11^C-acetate metabolic rate when compared to controls. By comparison, there was no corresponding increase in hepatic ^18^ F-FDG up-take in mice. This observation is similar to previous work comparing ^18^ F-FDG, 6-Deoxy-6-^18^ F-Fluoro-D-Glucose (^18^ F-6FDG) and ^11^C-acetate PET/CT imaging in a hepatitis viral infection-induced woodchuck model. Using tumor to liver uptake ratios, tumors were detected in 53 % of animals using ^18^ F-FDG, 0 % using ^18^ F-6FDG and 94 % using ^11^C-acetate [48]. These data are consistent with clinical observations of Cheung et al. which demonstrated that ^11^C-acetate was far superior to ^18^ F-FDG for HCC detection and staging in patients prior to liver resection or transplant [39].

Interestingly, the false negative rate of ^18^ F-FDG in MDR2^−/−^ mice is consistent with clinical evaluation of ^18^ F-FDG as the primary imaging biomarker in HCC in patients [37–40]. Moreover, recent quantitative studies of metabolic genes obtained from liver biopsies suggests that ^18^ F-FDG uptake in HCC is linked to expression of acetyl Coenzyme-A (CoA)-Synthetase-1 (ACSS1) an enzyme that generates acetyl-CoA from acetate and CoA, with the hydrolysis of ATP to AMP and diphosphate [49, 50]. In mice hepatic ACSS1 expression is relatively low with respect to other acetyl-CoA enzymes and in the absence of other acetyl-CoA synthesizing enzymes ACSS1 is not sufficient to support murine development [51]. The increase in cardiac ^18^ F-FDG in MDR2^−/−^ mice is not currently well understood, but may be linked to an increase in glucose transporters of the heart. It is well know that GLUT4, the main contributor of glucose transport in the heart [52] and MDR2 are both regulated by PI3K/AKT signaling pathway. PI3K-AKT controls the GLUT4 translocation from intracellular vesicles to the plasma membrane [53]. In a similar manner PI3K/AKT activity also regulates MDR2 transporter activity [54]. At this time no information exists pertaining to cardiac AKT levels in MDR2^−/−^ mice or the effect of MDR2 gene deletion on cardiac GLUT4 expression; however, current reports show that reduction of MDR activity with functional inhibitors increases chemo-sensitivity without affecting AKT phosphorylation [55]. The connection amongst MDR2, GLUT4, and ^18^ F-FDG in the heart requires additional investigation to better understand any interactions.

We are cognizant that observations in MDR2 gene knockout mice may not be 100 % comparable to that seen in patients. In MDR2^−/−^ mice chemotherapeutic clearance is potentially impaired, which would provide for a sustained dosing time [56]. MDR2^−/−^ mice have a significant impairment of ^99m^Tc-Sestamibi biliary excretion [57], which is a marker of MDR function, and thus biliary transport is reduced in MDR2^−/−^ mice. However, this does not appear to interfere with ^11^C-acetate PET/CT imaging which was clearly elevated in MDR2^−/−^ when compared to controls. To mitigate possible hepatobiliary change in ^11^C-acetate transport, standardized protocols could be used where treated and untreated groups are imaged longitudinally, thus increasing the statistical power and minimizing the possibility of erroneous results.

The second objective of this manuscript was to better understand the connection between lysophosphatidic acid (LPA) and HCC in MDR2^−/−^ model. Previous studies have identified the biosynthesis of LPA as a marker of HCC [58–60], and is known to be associated with tissue fibrosis [61–63] in this patient population. Moreover, we have previously shown that LPA variants were able to differentiate between liver disease and patients with HCC [59]. Interestingly, these data are consistent with our current, and previous [6], studies in MDR2^−/−^ mice where LPA is not only elevated contemporaneous with HCC, but the LPA 20:4 and 18:2 variants are highly consistent with data from the clinical populations with HCC. Currently the precise role and mechanism connecting LPA to HCC is not fully understood. However, LPA is known to be associated with endothelin derived growth receptors (EDGR) [64], and has previously been linked to cellular signaling molecules (cAMP) and pro-inflammatory cytokines (TNFα) [65, 66]. In MDR2^−/−^ mice with HCC there was a significant decrease in serum cAMP contemporaneous with an increase in LPA. The LPA receptor-3 (LPA3) is reported to be inhibitory of adenylate cyclase activity [64], and mice deficient in adenylate cyclase are desensitized to LPA [67]. Interestingly, reductions in cAMP have previously been reported in HCC [68]. Moreover, LPA has also been linked to the elevated expression of TNFα [69] which has been shown in patients with HCC [70]. In MDR2^−/−^ mice hepatic TNFα levels were increased concurrent with HCC and increased LPA. Combined, these data suggests that elevated LPA biosynthesis associated with HCC is potentially linked to modulations of cAMP and/or TNFα. These data clearly illustrate the multitude of pathways by which LPA can exert action within the oncogenic milieu. These interactions are exemplified by, *inter alia,* advanced glycation end products (RAGE) and hypoxia inducible factor 1 alpha (HIF1α) which are known to modulate cellular signaling associated with a variety of cancer types [71, 72]. In addition to binding to EDGR receptors, LPA has also been shown to bind to the receptor for RAGE [73], which is a key regulator of inflammation-associated liver carcinogenesis in MDR2^−/−^ mice [71]. Similarly, HIF1α has been shown to suppress apoptosis in HCC cells [72] and increases cellular responsiveness to LPA [74]. Taken overall, these data suggest that LPA plays a key role in the development and progression of HCC in MDR2^−/−^ and exerts its effects via a range of second messenger systems and/or modulation of cytokines and growth factors.

LPA variant analysis complements ^11^C-acetate PET/CT imaging. In MDR2^−/−^ mice changes in the LPA profile are contemporaneous with the emergence of HCC and in patients the LPA variant profile is corrected by liver transplantation [59]. The analysis is sensitive (nmol.ml), standard, inexpensive and can be incorporated in to a routine monitoring program to indicate the need for ^11^C-acetate PET/CT. Following LPA variant analysis ^11^C-acetate PET provides fmol/ml sensitivity and spatial information in a patient population which has non-diffuse disease, this would allow for ^90^Y thearaspheres or stereotactic body radiotherapy to be deployed as treatment of HCC.

## Conclusion

In conclusion, there is a need for additional preclinical studies in HCC to improve patient stratification and treatment options. In furtherance of this goal, the current manuscript details an evaluation of the MDR2^−/−^ murine model of HCC and provides an analysis of HCC emergence as monitored by serum (AFP, LPA and cAMP) and tissue (TNFα and ^11^C-acetate) markers. These data corroborate with previous clinical studies that show that serum AFP, LPA and ^11^C-acetate PET/CT studies are modulated following treatment [39, 46, 59] and supports future studies to identify novel chemotherapeutics targets for clinical trials. Going forward, and given the complexity of HCC, it is unlikely that any “single surrogate biomarker” (or target) identified above for HCC will provide a robust approach for monitoring disease and therapeutic response. Moreover, we believe that advancements in the field will rely on the strength of multiple markers in combination to provide the complete picture of disease progression and response to therapies. Finally, it is our current belief that the MDR2^−/−^ model in combination with validated HCC monitoring modalities will aid in these studies.
